# A Bibliometric Network Analysis of Coronavirus during the First Eight Months of COVID-19 in 2020

**DOI:** 10.3390/ijerph18030952

**Published:** 2021-01-22

**Authors:** Leonardo B. Furstenau, Bruna Rabaioli, Michele Kremer Sott, Danielli Cossul, Mariluza Sott Bender, Eduardo Moreno Júdice De Mattos Farina, Fabiano Novaes Barcellos Filho, Priscilla Paola Severo, Michael S. Dohan, Nicola Luigi Bragazzi

**Affiliations:** 1 Graduate Program of Industrial Systems and Processes, University of Santa Cruz do Sul, Santa Cruz do Sul 96816-501, Brazil; sott.mk@gmail.com; 2 Department of Medicine, University of Santa Cruz do Sul, Santa Cruz do Sul 96816-501, Brazil; brunarabbaioli@gmail.com; 3 Department of Psychology, University of Santa Cruz do Sul, Santa Cruz do Sul 96816-501, Brazil; daniellicossul@mx2.unisc.br; 4 Multiprofessional Residency Program in Urgency and Emergency, Santa Cruz Hospital, Santa Cruz do Sul 96810-072, Brazil; maribendersott@hotmail.com; 5 Scientific Writing Office Department, Higher School of Sciences of Santa Casa de Misericórdia de Vitória, Vitória 29025-023, Brazil; eduardofarina61@gmail.com (E.M.J.D.M.F.); fabiano.filho@edu.emescam.br (F.N.B.F.); 6 Graduate Program in Law, University of Santa Cruz do Sul, Santa Cruz do Sul 96816-501, Brazil; priscillasevero@hotmail.com; 7 Faculty of Business Administration, Lakehead University, Thunder Bay, ON P7B 5E1, Canada; msdohan@lakeheadu.ca; 8 Laboratory for Industrial and Applied Mathematics (LIAM), Department of Mathematics and Statistics, York University, Toronto, ON M3J 1P3, Canada

**Keywords:** COVID-19, coronavirus, pandemics, virus diseases, co-word analysis, strategic intelligence, bibliometric analysis, SciMAT

## Abstract

The COVID-19 pandemic has affected all aspects of society. Researchers worldwide have been working to provide new solutions to and better understanding of this coronavirus. In this research, our goal was to perform a Bibliometric Network Analysis (BNA) to investigate the strategic themes, thematic evolution structure and trends of coronavirus during the first eight months of COVID-19 in the Web of Science (WoS) database in 2020. To do this, 14,802 articles were analyzed, with the support of the SciMAT software. This analysis highlights 24 themes, of which 11 of the more important ones were discussed in-depth. The thematic evolution structure shows how the themes are evolving over time, and the most developed and future trends of coronavirus with focus on COVID-19 were visually depicted. The results of the strategic diagram highlight ‘CHLOROQUINE’, ‘ANXIETY’, ‘PREGNANCY’ and ‘ACUTE-RESPIRATORY-SYNDROME’, among others, as the clusters with the highest number of associated citations. The thematic evolution. structure presented two thematic areas: “Damage prevention and containment of COVID-19” and “Comorbidities and diseases caused by COVID-19”, which provides new perspectives and futures trends of the field. These results will form the basis for future research and guide decision-making in coronavirus focused on COVID-19 research and treatments.

## 1. Introduction

At the end of 2019, China detected a new type of coronavirus that led to a subsequent global pandemic. In February 2020, the World Health Organization (WHO) designated the disease, caused by the coronavirus SARS-CoV-2, as COVID-19 and a month later characterized it as a pandemic, which has resulted to date in 27 million confirmed cases and 900,000 deaths [1] and estimates of up to 1.2 million future deaths [2]. Currently the Weekly Epidemiological Update on COVID-19 published by WHO estimates more than 1,926,625 deaths and 88,828,387 confirmed cases [3]. In addition to the loss of life, the COVID-19 pandemic is directly affecting the global economy. Since it is too early to assess the final impact of this global recession, experience from previous pandemics suggests that each country will face different disruptive consequences and long-term effects due to their heterogeneous economic structure [4]. The global consequence of the pandemic has a ripple effect in economy from the primary to the tertiary sector. Research is showing the rapid growth in social burden, loss of income, unemployment, difficulty in accessing healthcare services and weakened educational and research systems at all levels [5].

A significant effort to research COVID-19 has been made in a short period of time. Scientific research is essential to evolve the knowledge and impacts of COVID-19 in order to control its massive effects, reduce infection rates and to develop methods of treatment, cure and contagious prevention [6,7]. Bibliometric Network Analysis (BNA) has been used by academics in order to support such issues, since it is a suitable approach to analyze a large amount of data by using advanced tools (e.g., Sci2tool, CiteSpace, VOSviewer, etc.). The results of BNA provides a holistic understanding of the efforts in multidisciplinary research to map publications, challenges, predict trends and critical points and measure the contribution of science derived from scientific works. The bibliometric approach contributes mainly to optimize the decision making of researchers and health managers [8].

Several bibliometric studies have been conducted in order to support research worldwide on COVID-19 (see Table 1, below). However, no study is known to have presented the strategic themes, thematic network structure and thematic evolution structure of coronavirus with focus on COVID-19 research during the first eight months in 2020, which can be identified using the SciMAT software. Besides, most of the studies do not perform the critical preprocessing step which can compromise the quality of bibliometric results. Moreover, most of them used CiteSpace and VOSviewer to perform analysis, and therefore is a lack of different point of views by in-depth studies of the most important topics, and identify trends along scientific advances supported by bibliometrics indicators, which can be achieved by using different and advanced bibliometric methods and tools such as SciMAT [9,10,11]. Besides, this analysis takes place under an interdisciplinary view, based on the Human, Biological and Exact Sciences, which enables a broader and deeper understanding of the pandemic impact on individuals. Therefore, our goal was to perform a BNA of coronavirus focused on COVID-19 from January until August 2020 in order to identify the most important themes, how such themes are evolving over time, and the hidden patterns in the network structure of all scientific works from the Web of Science (WoS).

## 2. Methodology and Dataset

BNA has been used to support researchers by enhancing the understanding of research fields from the discovery of relevant themes, evolution structure over time, trends, challenges and opportunities for the development of current and future works [36,37,38,39,40]. It also makes it possible to analyze the performance of a field of study in terms of scientific productivity (e.g., authors, countries, universities, etc.). This information helps in decision making both for the development of new theoretical and applied works and for the generation of knowledge, which can be used to support new research [41,42,43,44,45]. With this perspective in mind, our goal in this bibliometric study was to answer three main questions:

***RQ1:****What are the strategic themes of COVID-19 in 2020?*
***RQ2:****How is the thematic evolution structure of COVID-19 in 2020?*
***RQ3:****What are the trends and opportunities of COVID-19 for academics and practitioners?*

### 2.1. Methodology

In this research, a BNA was performed on the field of research of COVID-19 in 2020 by using the Science Mapping Analysis Software Tool (SciMAT). This tool was chosen due to the possibility to perform a complete science mapping analysis (from preprocessing to bibliometric network analysis), since allows a robust preprocessing step [41]. The BNA method merges the performance analysis approach with science mapping technique, which makes it possible to perform a broad scientific exploration into any research area using documents from scientific databases (e.g., Google Scholar, Scopus, Web of Science, among others) and provide an extensive analysis that can be used to move forward a higher perspective and knowledge of a specific field [39]. Such comprehension support decision-makers from governments, universities and industries interested in the area [46]. To perform this BNA followed four (4) steps were followed:

#### 2.1.1. Detection of Research Themes

The research themes were identified using a frequency and network reduction of words. The clustering algorithm used was the simple centers algorithm. To normalize data, the Salton’s Cosine was used to create the strategic diagram and the equivalence index was applied to normalize the co-word network of the thematic evolution structure.

#### 2.1.2. Depicting Research Themes and Thematic Network Structure

**Figure 1:**
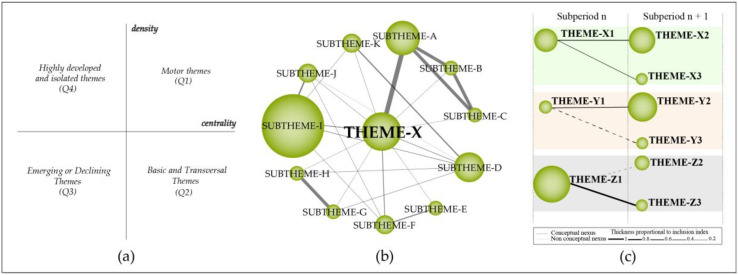
Strategic diagram (**a**). Thematic network structure (**b**). Thematic evolution structure (**c**).

The research themes were plotted in a bi-dimensional diagram (Figure 1a) composed of four quadrants, in which the ‘vertical-axis’ characterizes density (D.) and the horizontal-axis’ characterizes the centrality (C.) of the theme. The research themes were classified into four groups:

(a) Motor themes (1º quadrant—Q1): High centrality and density.
(b) Basic and transversal themes (2º quadrant—Q2): High centrality and low development.
(c) Emerging or declining themes (3º quadrant—Q3): Low centrality and density.
(d) Highly developed and isolated themes (4º quadrant—Q4): low centrality and high development.

#### 2.1.3. Detection of Thematic Areas

The thematic network structure (Figure 1b) characterizes the co-occurrence between the research themes and highlights the amount of relationships (C.) and internal strength among them (D.). The thematic evolution map (Figure 1c), provides an appropriate image of how the themes preserve a conceptual nexus throughout following subperiods. The size of the clusters is proportional to the number of core documents and the links indicate co-occurrence among the clusters. Solid lines indicate that clusters share the main theme, and dashed lines represent the shared cluster elements that are not the name of the themes. The thickness of the lines is proportional to the inclusion index, which indicates that the themes have elements in common.

#### 2.1.4. Performance Analysis

The scientific contribution was measured by analyzing the important research themes and thematic areas using h-index, sum of citations, core documents, centrality, density and nexus among themes.

### 2.2. Dataset

First, the search string that was used to explore the documents in the database was defined: (“Wuhan coronavirus” OR “Wuhan seafood market pneumonia virus” OR “COVID19*” OR “COVID-19*” OR “COVID-2019*” OR “coronavirus disease 2019” OR “SARS-CoV-2” OR “sars2” OR “2019-nCoV” OR “2019 novel coronavirus” OR “severe acute respiratory syndrome coronavirus 2” OR “2019 novel coronavirus infection” OR “coronavirus disease 2019” OR “coronavirus disease-19” OR “novel coronavirus” OR “coronavirus” OR “SARS-CoV-2019” OR “SARS-CoV-19” according to [31].

For the database, WoS was chosen because this database frequently indexes journals with higher impact factors if compared with others (e.g., Science Direct, Scopus, Google Scholar, etc). All of indexed databases within WoS were selected such as Science Citation Index (SCI-E), Social Science Citation Index (SSCI), among others. Only articles and reviews in English language were analyzed, therefore a filter was applied. 14,802 articles were obtained and exported from WoS, then imported into SciMAT software.

For the next step, a data preprocessing step was executed to exclude unwarranted information such as duplicate documents using Endnote deduplication module. Authors, and journals were excluded using SciMAT preprocessing tool. 26,679 words were extracted, and those with the same meaning were grouped (e.g., ‘SARS-CoV-2019’ and ‘SARS-CoV-19′; ‘PERSONAL-PROTECTIVE-EQUIPMENT’ and ‘PPE’ etc.). 168 broad words such as ‘HEALTH’ and search strings used in database such as ‘COVID-19′ were excluded to discover unidentified evidence. Finally, 26,511 words were included for BNA. Moreover, a preprocessing was also applied to correct and group articles according to publish date. For the strategic diagram analysis only one period was considered (January 2020–August 2020) and for the thematic evolution structure 3 different subperiods were considered: January until March (first subperiod), April, May, June (second subperiod), July until August (third subperiod).

**Figure 2:**
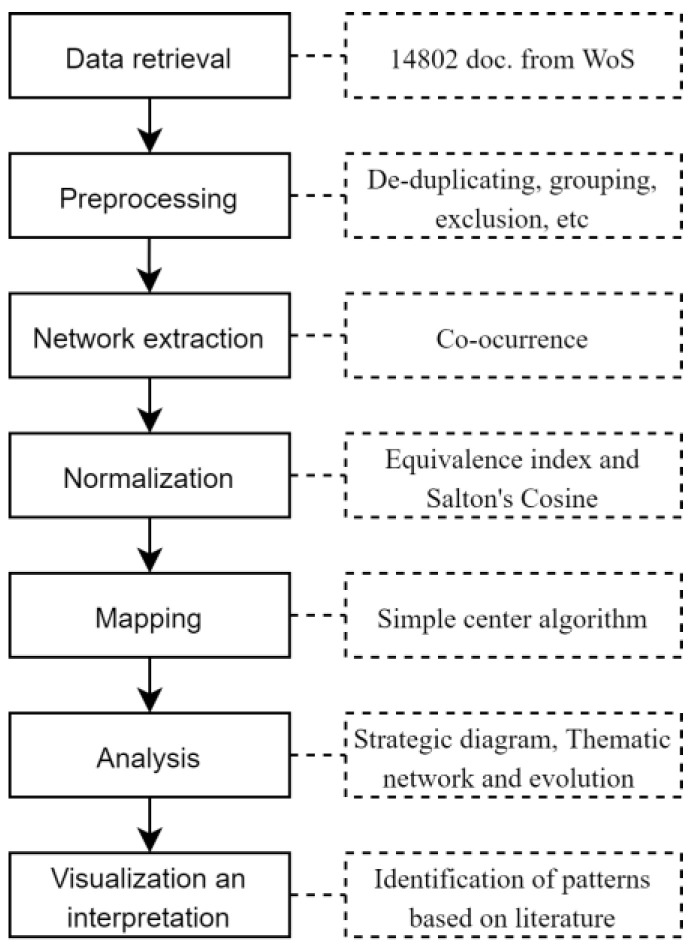
Workflow of the BNA.

Regarding the bibliometric software parameters, we considered specifically the authors’ words. Also, a data and network reduction were applied to extract the most important themes and exclude immature keywords and co-occurrences. For the network extraction co-occurrence among keywords was identified. For the normalization, Salton’s Cosine was used for the strategic diagram and the Equivalence Index for the thematic evolution structure. A simple center algorithm was used of mapping process. Finally, a core mapper was used, as well as h-index and sum citations. Figure 2 (below) shows the steps of the BNA performed in this research.

## 3. Bibliometric Network Analysis: Strategic Themes and Thematic Network Structure of COVID-19 in 2020

**Figure 3:**
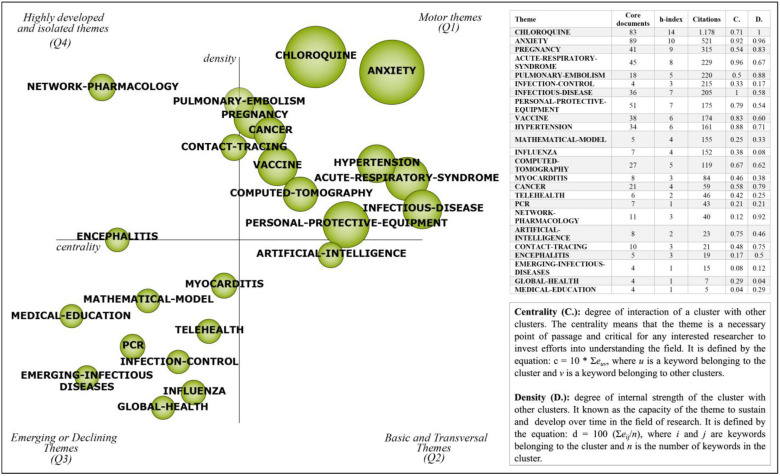
Strategic diagram depicting the performance of the research themes of COVID-19 in 2020.

**Figure 4:**
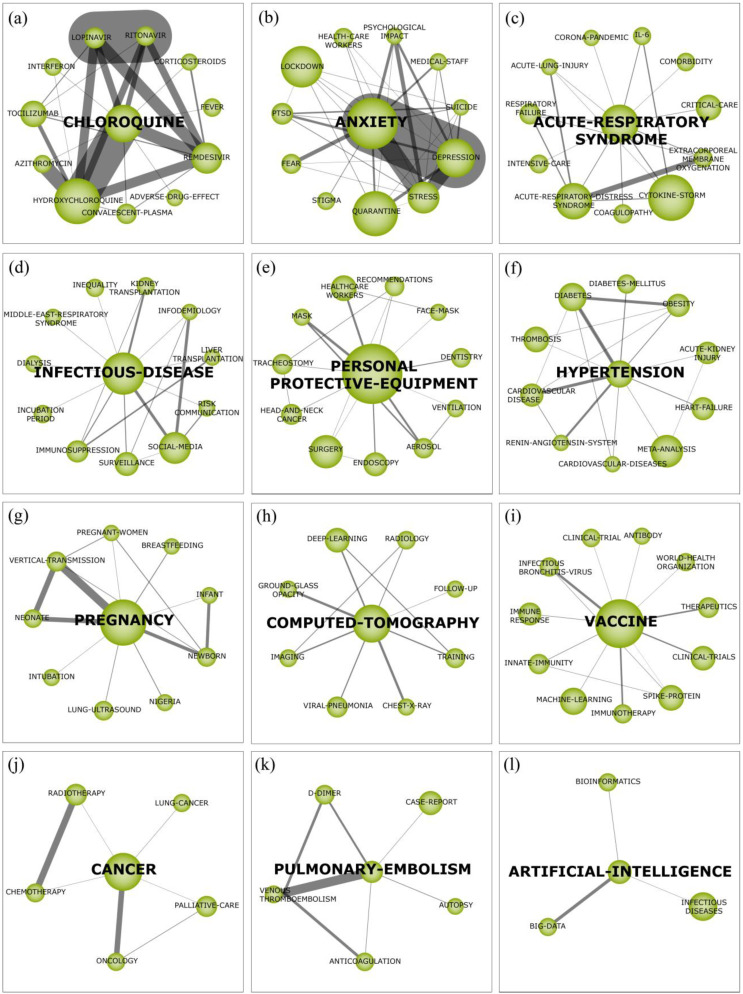
Thematic network structure of the strategic research themes of COVID-19 in 2020. (**a**) The cluster ‘CHLOROQUINE’. (**b**) The cluster ‘ANXIETY’. (**c**) The cluster ‘ACUTE-RESPIARTORY-SYNDROME’. (**d**) The cluster ‘INFECTIOUS-DISEASE(S)’. (**e**) The cluster ‘PERSONAL PROTECTIVE-EQUIPMENT’. (**f**) The cluster ‘HYPERTENSION’ (**g**) The cluster ‘PREGNANCY’. (**h**) The cluster ‘COMPUTED-TOMOGRAPHY’. (**i**) The cluster ‘VACCINE’ (**j**) The cluster ‘ONCOLOGY’. (**k**) The cluster ‘PULMONARY-EMBOLISM’. (**l**) The cluster ‘ARTIFICIAL-INTELLIGENCE’.

In this section, the results of the BNA of COVID-19 in 2020 as well as an in-depth and exhaustive investigation of the strategic themes of COVID-19 in 2020 are presented (see Figure 3, below). The thematic network structure of each cluster can be visualized in (Figure 4 below) which provides a good representation of the co-occurrence among keywords and allowing the depiction of hidden patterns.

Composed of four quadrants, the strategic diagram presents 24 clusters, of which 11 are motor themes, one is basic and transversal, 10 are emerging or declining themes and only two are highly developed and isolated themes. The size of the clusters represents the number of associated documents. Core documents, h-index, citations, centrality (C) and density (D) are presented for each cluster (see Figure 3, below). The ‘ANXIETY’ cluster contains the largest number of associated documents (89) and has the highest combination of density and centrality. Although several clusters have representativeness in the field of study, the ‘CHLOROQUINE’ cluster stands out as the most cited topic in the period (1178) and is the first ranked in terms of h-index and density. In addition, the ‘INFECTIOUS-DISEASE’ theme is the first ranked in terms of centrality.

A broad look allows us to see that the motor themes are related to research that seeks a solution to the problems of the pandemic, such as discussions about medicines, vaccines, and symptoms. In contrast, the emerging or declining themes discuss emerging infectious diseases, mathematic models, issues related to global health since many works have studied different regions or presented pandemic behavior worldwide, and changes in the health sector linked to clusters ‘TELEHEALTH’ and ‘MEDICAL-EDUCATION’. The motor themes that drive the discussions in the COVID-19 field will be explored in depth in the next section.

### 3.1. Chloroquine

The cluster ‘CHLOROQUINE’ (see Figure 4a, below) is strongly interconnected with sub-themes related to drugs tested to combat COVID-19. The connection between ‘CHLOROQUINE’ and ‘HYDROXYCHLOROQUINE’ is explained by their similar effect on the virus, since the mechanism of action of the two molecules is identical [47]. Chloroquine and hydroxychloroquine have no results that suggest them becoming a standard treatment for COVID-19, due to the lack of high quality randomized controlled trials. Although some studies recommend its use [47], others do not indicate based on the explanation that the use does not differ in the clinical course of the disease [48]. The co-administration of hydroxychloroquine with ‘AZITHROMYCIN’ has been tested in several studies trying to prove that azithromycin reinforces the effect of hydroxychloroquine when used concomitantly, which explains the cluster correlation [49].

Furthermore, the correlation between ‘LOPINAVIR’ and ‘RITONAVIR’ is explained by studies that combined these drugs in a fixed dose as a treatment option for COVID-19, because patients with severe form of COVID-19 treated with this therapy had a significant increase in the levels of hepatobiliary and pancreatic plasma markers, in addition to gastrointestinal effects. From this, it is suggested that this adjunct therapy of routine pneumonia medications be applied to COVID-19 patients [50]. Also, ‘REMDESIVIR’ is a prominent antiviral which has in vivo evidence studies of the potential utility in the treatment of patients with MERS-CoV [51] soon after the onset of symptoms or in severe cases where it occurs prolonged virus replication [52]. According to recent research, patients who received remdesivir had a numerically shorter time to clinical improvement than those who received placebo, although confirmation in larger studies is still needed [53]. In this sense, the relationship between chloroquine and hydroxychloroquine with lopinavir/ritonavir and remdesivir can be explained by the concomitant adverse effects of these drugs, such as the prolongation of the QT interval on the electrocardiogram and hypoglycemia [54].

In addition, the IL-6 interleukin antagonist ‘TOCILIZUMAB’ may be an important drug, as it has been shown to be effective in blocking the febrile and inflammatory response of the storm associated with IL-6 as shown in the following study [55]. For this reason, it stands out in the cluster since the cytokine release syndrome (CRS) seems to play an important role in severe cases of COVID-19 and IL-6 is the key molecule of this phenomenon.

Also, ‘INTERFERONS’ (IFN) type I is currently being evaluated in a clinical trial to treat MERS-CoV and have gained prominence for the treatment of COVID-19. In Vitro, SARS-CoV-2 replication is inhibited by IFN-α and IFN-β at concentrations that are clinically achievable in patients [56]. The combination of IFN-I with lopinavir/ritonavir, ribavirin or remdesivir can improve its effectiveness, due to the efficiency of such combinations observed In Vitro in other coronaviruses [57]. In addition, it is estimated that the safety and efficacy of ‘CORTICOSTEROIDS’ dexamethasone at a dose of 6 mg (oral or intravenous) once daily and its preliminary data suggested a 35% reduction in the risk of death, that is, an improvement in survival in COVID-19 [58].

In this sense, all drug studies as a possible treatment for the effects of this new disease are necessary because they play an important role in fighting the pandemic and reducing mortality. There is still no standard treatment for COVID-19 and currently each country has its own guidelines according to evidence-based protocols, which are updated day after day according to the evolution of scientific research.

### 3.2. Anxiety

The cluster ‘ANXIETY’ (see Figure 4b, above) demonstrates that researchers have put their efforts into studies related to symptoms and psychological disorders, since the pandemic caused by the coronavirus puts public health policies under discussion. This is due to the high demand for care that those infected by the virus need, as well as investments in disease prevention. In addition, it has caused psychological distress and fear in several individuals, mainly due to hospitalization, isolation and quarantine periods. In this sense, researchers are investigating the issues of ‘ANXIETY’ [59], ‘STRESS’ and ‘DEPRESSION’ [60] in the general population, in children and adolescents [61] and pregnant women [62]. Also, studies on ‘QUARANTINE’ and ‘LOCKDOWN’ investigated the lifestyle and changes that occurred during the pandemic, as well as the related psychic effects [63,64].

Current evidences show that the psychological distress of patients with COVID-19 demonstrate feelings of guilt, helplessness and fear of the uncertainty of disease progression, as well as depressive symptoms, which may be related to inflammatory markers, affecting the severity of the disease [65,66]. Besides that, professionals working on the front line of coping with coronavirus may also have their mental health affected. It was confirmed that a large number of these professionals have experienced mood and sleep disorders during the pandemic, which can be a risk to mental health [67]. However, despite the large number of researches carried out on the pandemic effects on the subjects’ mental health, it is also necessary to conduct longitudinal studies in the future, in order to identify the influence of this psychological suffering on long-term mental health [68].

### 3.3. Acute Respiratory Syndrome

The cluster ‘ACUTE-RESPIARTORY-SYNDROME’ (see Figure 4c) is a motor theme with high density and centrality because it is the main severe clinical presentation of the COVID-19 disease. It is related to ‘INTENSIVE-CARE’ and ‘CRITICAL-CARE’ due to patients presenting that condition usually needs to be treated in Intensive Care Unit (ICU) [69]. Also, it might have as treatment option the use of ‘EXTRACORPOREAL-MEMBRANE-OXYGENATION’ due to ‘ACUTE-LUNG-INJURY’ and ‘RESPIRATORY-FAILURE’. The use of extracorporeal membrane oxygenation (ECMO) is highly related to ‘ACUTE-RESPIRATORY-DISTRESS-SYNDROME’ that can be understood as a more severe form and a characterization of acute-lung injury in patients with acute respiratory syndrome, because it is a good option for its treatment [70]. The density of the ‘CYTOKINE-STORM’ cluster and its relation to acute respiratory syndrome and ARDS can be interpreted by virtue of the amount of research made to elucidate the severity of the lung injury in COVID-19 patients caused by an inflammation stress in their bodies related to interleukins such as ‘IL-6′ [71]. IL-6 is also higher at patients that requires ICU admission [71]. ‘COMORBIDITY’ is another acute respiratory syndrome related cluster due to the evidence of correlation between previous comorbidities in a group of patients and the severity of the Sars-Cov2 infection [72].

### 3.4. Infectious Disease

Coronavirus have caused three serious human ‘INFECTIOUS-DISEASE(S)’ (see Figure 4d, above) called Severe Acute Respiratory Syndrome (SARS) in less than 20 years, which globally demonstrates the great need for planning and implementing comprehensive policies to prevent pandemics. The strong relationship between the infectious disease and ‘SOCIAL-MEDIA’ clusters is explained by the important role that rapid communication among countries and population plays in preventing the spread of COVID-19. Another important field of study in viral outbreaks is ‘INFODEMIOLOGY’, that is related to the distribution of determinants of information mainly online to inform and improve public health. It is known that health information available online can influence health behavior of the population. In addition, the digital epidemiology policy can be used to monitor disease outbreaks [73]. All of these measures improve ‘SURVEILLANCE’ by containing the disease, detecting cases and generating statistics with characteristics of the disease, in order to identify the ‘INCUBATION-PERIOD’ of the SARS [74].

The COVID-19 pandemic also brought into discussion the subthemes ‘KIDNEY-TRANSPLANTATION’ and ‘LIVER-TRANSPLANTATION’. Transplanting patients puts them at risk of contagion of COVID-19 during an ‘IMMUNOSUPPRESSION’ discharge period, however postponing a kidney transplant, for example, also puts the patient at risk for the lethality of ‘DIALYSIS’ they may need to undergo. The difficulty of this decision is accentuated by the lack of evidence-based algorithms to guide the practice and that is why transplant centers are having to assess the risk on their own [75]. The decrease in transplants during this period is explained by the lack of ICU beds and the concern of infection by both donor and recipient. For transplants performed, COVID-19 tests are indicated, as post-transplant immunosuppression may allow uncontrolled viral proliferation in undiagnosed patients [76].

### 3.5. Personal Protective Equipment

The cluster ‘PERSONAL-PROTECTIVE-EQUIPMENT’ (see Figure 4e) has expressive relationships with ‘HEALTHCARE-WORKERS’, ‘MASK’ and ‘AEROSOL’ subthemes. Face masks are being used worldwide as a strategy to contain COVID-19 infection which occur due to inter-human transmission, mainly through droplets and viral particles of ‘AEROSOL’ [77,78]. Other forms of propagation are: body fluids, such as feces, blood and urine [77]. Psychologically, the mask represents security, as it signals a reduction in the possibilities of contamination [79]. However, for those who ignore social distancing and hand washing, this security tends to be flawed, since the two aspects identified are considered more effective for prevention [79].

For the general population, doubts are still recurring about who really needs to wear a mask and which model is more appropriate [79]. In view of the scarcity of resources, fabric masks, simple, inexpensive and sometimes without proof of effectiveness, are being used in masse as a precautionary principle [80]. In relation to ‘HEALTHCARE-WORKERS’, ‘FACE-MASK’ is considered as a complete facial protection and are associated with disposable insulating clothing, in order to guarantee higher protection, especially in red zones from those heavily infected [81]. In addition, researchers highlight skin irritation and physical exhaustion as some damage caused by prolonged use of individual protection tools [81,82].

In terms of production, the cluster that stands out the most is ‘SURGERY’ which is associated with ‘ENDOSCOPY’. Regarding this relationship, trans nasal surgery is a potential transmitter of the virus, since the maneuvers used come in direct contact with the patient’s respiratory mucosa [78]. In this perspective, the scope of contamination possibilities in the operating room must be observed, such as the identification of contaminated fluids in the surgeon’s arm, in the surgeon’s abdomen and at the foot of the bed [83]. In addition, the performance of an endoscopy anticipates the identification of patients at risk, infected with the coronavirus [84]. Thereafter, rapid or seventy-two-hour viral tests are applied, however, it must be considered that infections by the virus may not be detected through serological tests [85].

### 3.6. Hypertension

The cluster ‘HYPERTENSION’ (see Figure 4f, above) stands out considering that during the COVID-19 pandemic, comorbidities were present in almost half of hospitalized patients, being the most prevalent and ‘DIABETES’ as the second one [86]. In addition, considering the number of diabetics’ obese people, the correlation between the subthemes ‘DIABETES’ and ‘OBESITY’ occurs because the latter one is a confirmed cause of the former one. Also, diabetic people are at higher risk of contracting acute and chronic infections, as they are affected by low-grade chronic inflammation that can facilitate cytokine storms and contribute to higher mortality and worse outcomes with COVID-19 [86].

Furthermore, the relationship between the cluster and the subthemes ‘CARDIOVASCULAR-DISEASE’ and ‘RENIN-ANGIOTENSIN-SYSTEM’ occurs because studies involving hypertensive patients with COVID-19 show that renin and angiotensin inhibitors improve the clinical picture and reduce the mortality of patients hypertensive with COVID-19, considering the researchers’ suggestions that these patients could benefit from the persistent use of angiotensin-converting enzyme inhibitors (ACEIs) or angiotensin receptor blockers (BRAs) for antihypertensive treatment [87]. Besides that, the ‘THROMBOSIS’ subtheme also stands out as an important topic in patients with COVID-19. These studies indicate that coagulopathy associated with COVID-19 is the result of an inflammatory response to SARS-CoV-2 infection, which results in inflammation of the thrombus and thrombosis, especially in more severe cases [88].

### 3.7. Pregnancy

By analyzing the cluster ‘PREGNANCY’ (see Figure 4g, above), studies indicate concerns about the contagion of ‘PREGNANT-WOMEN’ by the coronavirus, mainly because it is a little-known virus and with no evidence about the risks of congenital malformation or abortion [89]. Given the small number of ‘NEWBORNS’/‘NEONATE’ positive for COVID-19 worldwide, recent research has discussed whether there is a possibility of ‘VERTICAL-TRANSMISSION’. The clinical presentation of the disease and the nonspecific results of babies’ laboratory tests suggest that mother could directly transmit the virus to their offspring [90]. In this perspective, research efforts has been used to understand the transmission of the virus through ‘BREASTFEEDING’ and, for this reason it is indicated that mothers with COVID-19 should not breastfeed until their full recovery [91].

### 3.8. Computed Tomography

The cluster ‘COMPUTED-TOMOGRAPHY’ (see Figure 4h, above) is strongly associated to other themes related to medical image such as ‘RADIOLOGY’, ‘CHEST X-RAY’, ‘IMAGING’ and ‘GROUND-GLASS OPACITY’. Radiology is the medical field that studies types of medical imaging exams like chest X-rays and computed tomography. Since COVID-19 is a cause of severe acute respiratory syndrome, lung image examination is necessary to evaluate the patient lung status, hence, ‘COMPUTED-TOMOGRAPHY’ is the most dense and central cluster between all medical imaging exams because it is the first option to evaluate lung diseases in emergency scenarios. Ground glass opacities is a common initial image finding in most cases [92]. The other clusters ‘DEEP LEARNING’ and ‘TRAINING’ are related to computed-tomography. A large amount of papers were published during the COVID-19 pandemic using convolutional neural networks to recognize Sars-Cov2 infection in medical images [93], which requires training the algorithms on medical data.

### 3.9. Vaccine

The cluster ‘VACCINE’ (see Figure 4i, above) is considered a motor theme since it has high density and is highly centralized has a strong relation to the cluster ‘INFECTIOUS-BROCHITIS-VIRUS’ since it is the name given to a previous infectious disease caused by another coronavirus [94], not the SarsCov 2, and it could probably serve as prototype to make vaccines based on virus proteins such as a ‘SPIKE-PROTEIN’ [95,96], one of the others related clusters, to help with the pandemic control. The ‘VACCINE’ cluster is also related to ‘CLINICAL-TRIAL’, ‘ANTIBODY’, ‘IMMUNE RESPONSE’, ‘INNATE-IMMUNITY’ and ‘IMMUNOTHERAPY’. To deliver a vaccine to the market and to validate its clinical efficiency there is a need to conduct a clinical trial and evaluate the immune response through the antibody serum levels. This can be understood as a kind of immunotherapy. The individual response to a vaccine and the development of high serum levels of antibodies is associated to its innate immunity, and it remains a challenge for vaccinology to predict it [97]. ‘MACHINE-LEARNING’ is another cluster related to ‘VACCINE’ owing the fact that it could probably help in predicting individual response of the vaccine and maybe either predicting which virus protein would cause more immunological activation.

### 3.10. Cancer

The cluster ‘ONCOLOGY’ (see Figure 4j, above) presents discussions about treatments for patients during the pandemic caused by the worldwide spread of coronavirus. Cancer patients are more vulnerable to the virus due to their already poor health and weakened immunity, in addition to comorbidities and use of immunosuppressive drugs [98]. For instance, the consequences of COVID-19 are greater in patients with ‘LUNG-CANCER’ [99]. In this context, several call centers were reorganized due to the contamination of professionals and the reduced supply of personal protective equipment. As a result, the number of treatments involving ‘RADIOTHERAPY’ and ‘CHEMOTHERAPY’ was reduced, as new investigations were postponed and even when the treatment continued, it was modified, with the switch from intravenous to subcutaneous or oral medications, which is shorter in administration. However, despite the fact that cancer treatment is invasive, it does not exponentially increase the risk of serious infection by COVID-19, and treatment should be maintained, even if in a palliative way [100] with all possible protective measures [101].

Moreover, the pandemic highlighted the worldwide difficulty of integrating the perspectives of ‘PALLIATIVE-CARE’, cancer treatment and public health. Social inequalities in access to health services, sanitation and possibilities for individual and community protection also became clearer. Groups that are more likely to contamination and death by the coronavirus are those who are poor, who live in areas with a higher population density, with less comprehensive and organized health systems, as well as the elderly, migrants, homeless people and people with disabilities [102].

### 3.11. Pulmonary Embolism

The cluster ‘PULMONARY-EMBOLISM’ (see Figure 4k, above) stands out because pulmonary dissections of patients treated with COVID-19 revealed this pathology. In this sense, critically ill patients with COVID-19 admitted to the ICU have a greater state of hypercoagulation and a greater likelihood of ‘VENOUS-THROMBOEMBOLISM’ complications due to the hypoxia caused by pneumonia. In addition, an inflammatory and infectious state causes endothelial dysfunction, increased thrombin, and reduced fibrinolysis. These pathophysiological aspects reveal the importance of an early diagnostic suspicion of thromboembolism and the initiation of prophylaxis or ‘ANTICOAGULATION’ therapy, especially in patients with worsening dyspnea [103]. In fact, it was identified factors for the highest chances of hospital death in COVID-19 patients, which are advanced age, SOFA score 0 and, especially in the cluster, ‘D-DIMER’ level > 1 μg/mL, which explains the focus of this subtheme, since this biochemical is elevated in cases of pulmonary thromboembolism [104].

### 3.12. Artificial Intelligence

Although the cluster ‘ARTIFICIAL-INTELLIGENCE’ (see Figure 4l, above) is not a motor theme, it appears to be a technology that has been widely applied to COVID-19. It has a strong relation to ‘BIG-DATA’, since most of artificial intelligence (AI) techniques are machine and deep learning, therefore they need historic data to learn the patterns. ‘BIOINFORMATICS’ is related to ‘ARTIFICIAL-INTELLIGENCE’ cluster probably owing the fact that the data to train AI models need to be digitalized and available in databases. ‘INFECTIOUS-DISEASES’ appears here as a related cluster mainly because the scope of the study is an infectious disease (COVID-19) and AI can improve predict diagnosis and prognosis of this variety of infection [105]. AI has had many applications in the coronavirus pandemic including segmentation and diagnosis of COVID-19 in medical images [106], forecasting [107], diagnosis in the emergency care with unspecific laboratory tests [108] and many more. The pandemic has shown the medical community the need to improve AI related research and guidelines for it have been released [109,110] and will probably implicate in future works.

## 4. Thematic Evolution Structure of COVID-19 in 2020

**Figure 5:**
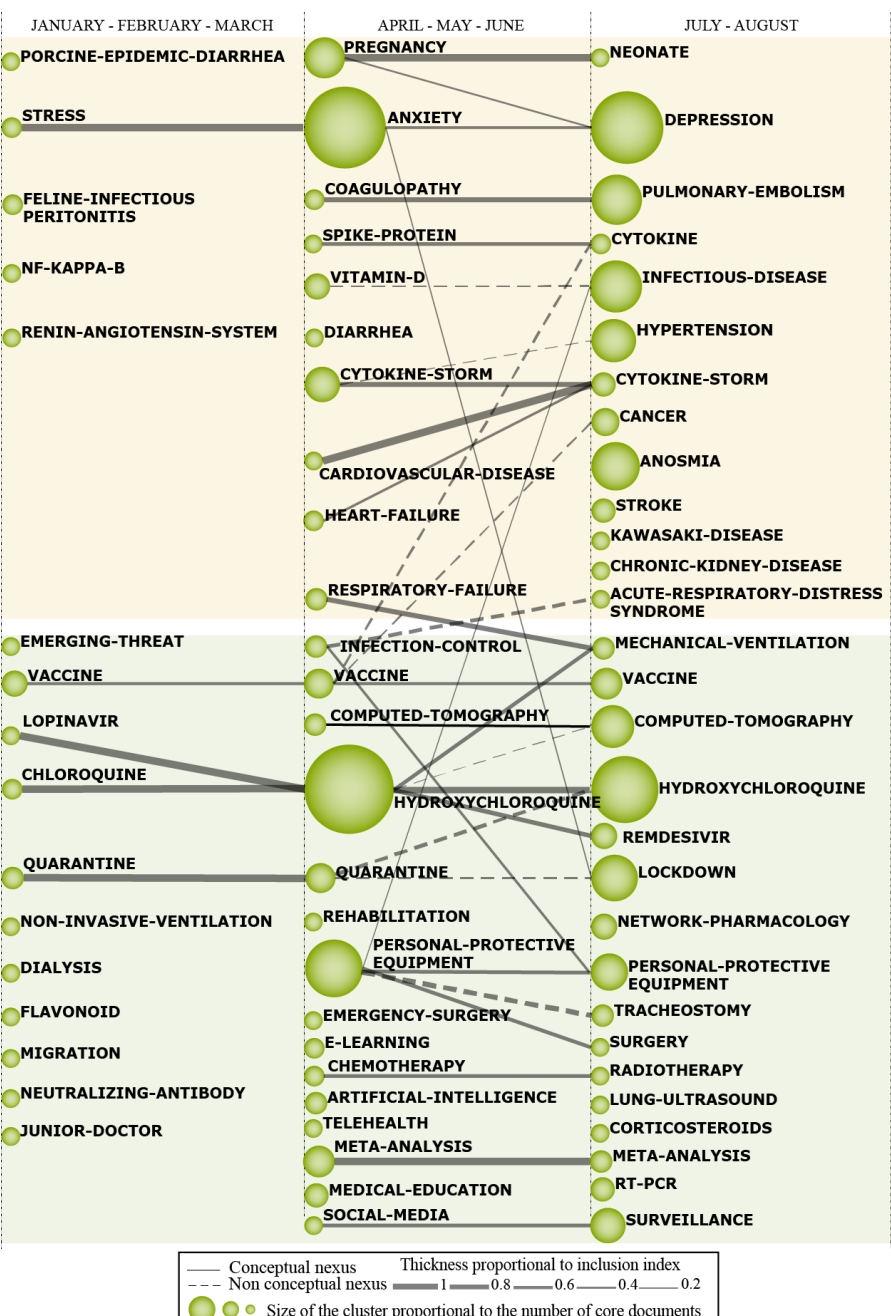
Thematic evolution structure of covid-19 from January to August in 2020.

The analysis of the thematic evolution of themes inherent in COVID-19 research (see Figure 5, below) contributes to the analysis of the most prevalent concepts and those that are emerging and their connections over the years. Thematic evolution map is read in the following language: solid lines refer to linked clusters that share a main item; dotted line refers to themes that share components that are not the main item; thickness of the edges is proportional to the inclusion index; and the dimensions of the spheres is proportional to the number of published documents associated with each cluster [41].

This evolution map was divided into three subperiods, namely: January-February-March; April-May-June; and July-August. In addition, it has two main thematic areas: Comorbidities and diseases caused by COVID-19 and Damage prevention and containment of COVID-19. The first (orange color) field covers 28 clusters aimed at comorbidities and disease caused by COVID-19, among these, eight show individual performance, that is, disconnected from other clusters. The second (green color) field covers 48 clusters related to damage containment techniques caused by the incidence of the pandemic COVID-19, among them, 17 present themselves in isolation.

### 4.1. Comorbidities and Diseases Caused by COVID-19

The first subperiod (January-February-March) is characterized by scarcity in scientific production, compared to the other periods. The cluster ‘STRESS’ is the motor theme as it establishes strong connections with ‘ANXIETY’ and ‘DEPRESSION’ in the second and third periods. These themes have as a common characteristic due to the fact that they are subjective dysfunctions and/or pathologies. In this sense, it is possible to understand that the COVID-19 pandemic unbalances meanings, triggers questions about the attribution of values, beliefs and desires. The April-May-June subperiod includes the ‘ANXIETY’ cluster as the most prominent, since it has a high number of associated documents. This theme is associated with ‘LOCKDOWN’, which expresses the effects of the required adaptations of routines. These sudden changes demand a subjective process of producing meanings and reframing.

The themes ‘ANXIETY’ and ‘DEPRESSION’ are related to psychological disorders, which may occur concurrently or consecutively, and are strongly influenced by the level of ‘STRESS’ experienced. They are caused by the psychological pressure exerted by the pandemic on individuals. In this perspective, the first study on the prevalence of depressive symptoms in the quarantined population in Shenzhen identified an incidence of 6.21% of depressive symptoms in this population, making it possible to associate demographic and clinical variables, such as age, marriage and education level, for greater propensity to the appearance of these symptoms [60]. Another study also identified the existence of a high level of depression in patients with COVID-19 [66]. In addition, the level of anxiety is around 30% among family members of health professionals, and the appropriate treatment for this population is crucial [59].

The clusters ‘PREGNANCY’ and ‘NEONATE’, respectively from the second and third periods, are strongly related since, for the development of the studies, they share main and guiding items. The greatest concern of researchers on this topic refers to the incipience of research on the implications that contamination by coronavirus can have on pregnant women and their newborns, mainly because it is an unknown virus and that can have long-term effects.

In this perspective, there are: ‘COAGULOPATHY’ and ‘PULMONARY-EMBOLISM’; ‘CARDIOVASCULAR-DISEASE’ and ‘CYTOKINE-STORM’. Hypoxia in severe COVID-19 patients can increase blood viscosity and stimulate thrombosis. Furthermore, the infection can induce dysfunction of endothelial cells, generate excess thrombin and promote the shutdown of fibrinolysis, characterizing a state of hypercoagulability [111]. Intravascular pulmonary coagulopathy also has implications for the understanding of cardiovascular mortality, since the immunothrombotic pathology will be aggravated by cardiac ischemia that accompanies the development of Acute Adult Respiratory Syndrome [112]. Patients can develop edema and lung failure and develop damage to the heart, liver and kidneys, all due to a storm of cytokines that cause systemic inflammatory symptoms [113].

### 4.2. Damage Prevention and Containment of COVID-19

This thematic area provides the visualization of the main methodological strategies adopted for the prevention and containment of damages caused by the pandemic COVID-19 in 2020. In this sense, the ‘VACCINE’ cluster appears successively during the three subperiods since the running to develop an effective vaccine seems to be the most reasonable strategy to contain the virus according to previous pandemic situations. In this same perspective, there is an association between the themes: ‘CHLOROQUINE’, from the first subperiod and ‘HYDROXYCHLOROQUINE’ from the second and third subperiods since the scientific community was studying and testing the medicine in order to understand its effective to prevent the virus. There are efforts to find antiviral activity in chloroquine since the 1950s [114] and there was never much benefit on it. Once the Sars-Cov2 pandemic started, scientists all over the world have focused their efforts on finding a drug to cure COVID-19 and since chloroquine and hydroxychloroquine, a similar compound but with usually less side effects, are cheap and are safe for human use, researchers tried to use it in patients infected with the disease. After a questionable non-randomized open label clinical trial that reached the suspicious conclusion that hydroxychloroquine and azithromycin [115] together were effective in coronavirus elimination from nasopharyngeal swabs, a huge amount of other studies started to take place, despite the fact that hydroxychloroquine treatment had a low pretest likelihood of being effective at reducing disease morbidity and mortality.

The ‘QUARANTINE’ cluster comprises the first two sub-periods, and finally, it evolves to ‘LOCKDOWN’, as this strategy has been used in order to reduce infections and dissemination of the virus. Consistently, the main theoretical-scientific model used for data collection and interpretation was ‘META-ANALYSIS’, which is found in the second and third subperiods.

The April-May-June and July-August subperiod include ‘PERSONAL-PROTECTIVE-EQUIPMENT’, which represents a strongly developed theme, since it presents a high number of associated documents and co-occurrence with other clusters, namely: ‘TRACHEOSTOMY’ and ‘SURGERY’. Regarding these crossings, the importance of tracheostomy to assist in weaning patients who are 7 to 10 days in invasive mechanical ventilation is highlighted. This surgical procedure offers a sealed system for continuous ventilatory support and allows a lesser need for patient sedation [116]. Therefore, there is a need for adequate protocols and personal protective equipment to protect health professionals from the threat of infection.

Another important fact is that every viral disease, that has as its most common target the lung system, provoking acute lung injury, never had a specific curative treatment since it was only support care using non-specific drugs such as ‘CORTICOSTEROIDS’ and invasive ‘MECHANICAL-VENTILATION’ for those patients with respiratory failure. This might be the reason that these clusters are more common in the July-August period, when the scientific community started to realize that the acute lung injury caused by COVID-19 should be treated as other viral pneumonias and researchers should not keep seeking for new virus specific treatments.

## 5. Comparison of Results and Suggestions for Future Work

Bibliometric analysis providing scientific evolution of COVID-19 were suggested in literature [117]. The results of such analysis showed four thematic areas: “health and pandemic management”, “the disease and its pathophysiology”, “clinical epidemiology of the disease” and “treatment of the disease”. Our results showed similar results through the strategic diagram and the scientific evolution and found two major thematic areas, related to research that seeks a solution to the problems of the pandemic and that discuss emerging infectious diseases, mathematical models and related issues to global health. The similarity of results comes due to the fact that the four thematic areas of the article are included in our motor themes, which have been depicted and studied separately for a better analysis of the thematic evolution and trends of COVID-19. The results of scientific evolution make it possible to form government and business policies based on evidence, in addition to helping to combat incorrect data.

Our study differs from other bibliometric analysis by revealing important trends. For instance, the cluster ‘ANXIETY’ (see Figure 4b, above) highlighted mental health as a relevant post-pandemic impact. Behavioral alterations, phobias, stress, anxiety and depression should be highlighted in scientific studies for the general population and for health professionals working on the front line. In this perspective, it can be predicted that future work will be focused on mental and psychological aspects, since our results demonstrate such problems will be a hotspot due to the current lack of resources such as capital, wealth, job opportunities, among others.

This research also differs from a bibliometric study published in May 2020, which used the SciMAT software to analyze publications from 1970 to 2020 [32] in order to obtain a conceptual analysis of the types and strains of coronavirus in the literature. This article, on the other hand, aimed to provide a more focused and updated view of the field of study. Although there is a great relevance of exploring the past to have reference on how to act in the present is important, but our results present a up to date data that were not included in the study abovementioned and such knowledge can certainly complement future research. This article analyzed publications from January to August 2020, thus, new research will be carried out to include the coming months of the pandemic, in order to provide updated analyzes and new points of view on the topic.

Despite initial studies on the different strains of the coronavirus dating back to 1970 [6,13,22,31], there has recently been a significant increase in research related to the field (see Table 1). This large number of works published in a short period denote the researchers’ concern with pandemic and its physical and psychological effects for individuals, especially for groups at risk. The analyzed papers present discussions about quarantine and social isolation, which can generate anxious and depressed symptoms and even trigger psychological disorders. Several studies present data on the ineffectiveness of medications such as chloroquine and hydroxychloroquine, which were widely prescribed, especially in the beginning of pandemic, as a form of treatment for COVID-19.

In addition, the massive purchase of medical, food and hygiene supplies in several countries around the world can be considered as a form of neurosis, which arises from the search for ego drive satisfaction, to the detriment of collective thinking. In this sense, the pandemic creates and recreates meanings of collectivism and individuality, which are signified and re-signified by the subjects. In this context, a collective feeling of uncertainty about the future and about the physical, social, emotional and psychological impacts of illness is created. Human beings never wanted social interaction as much as during quarantine and lockdown. When social media threatened real social interactions, individuals began to have these media as the only alternative, starting to value lost social contact. Therefore, pandemic, even after vaccination and the decrease in the number of active cases of COVID-19 in the world, will continue to show traces in the interpersonal relationships and mental health of individuals.

The scientific evidence about a behavioral pattern that expresses the effects of social isolation and loneliness related to mental health problems. Data generated in the United Kingdom during the pandemic indicate that, although increased symptoms of anxiety and stress are expected responses in the population during periods of confinement, people with this medical history are more likely to have self-destructive behaviors, such as self-mutilation and/or suicide [118]. Therefore, it is still reasonable to suggest future work related to psychological aspects to deal with the consequences of the coronavirus.

Although the AI cluster is not considered as a motor theme (see Figure 4l, above), we still highlight its importance. The research regarding the use of AI technologies for better diagnosis, prognosis and control of the coronavirus outbreak during 2020 have made significant improvements to the spread the knowledge and the importance about its technology in the healthcare community. Although there have been thousands of studies using AI during the pandemic, it still lacks external validation of any impact on clinical practice in real life. A study from September 2020 showed that there were only 64 AI algorithms approved by the FDA and none of them were strictly used for COVID-19 purposes [119]. However, in 2020 the CONSORT-AI and SPIRIT-AI, guidelines were published with recommendations on how to proceed with trials that utilize AI and how to better report on it in articles [109], so the healthcare community should expect in the next years more trials to validate AI tools and get the results applicable to real life and clinical practice.

From the results of this BNA, we realized the global urgency to carry out studies that enable greater knowledge about the new virus and its treatment possibilities. Several randomized clinical trials with a control group are underway to test different drugs, however, none have demonstrated sufficient safety and efficacy for standard treatment. We conclude that the numerous studies with hydroxychloroquine show the need that the current state of research has in having more comprehensive analyzes to enable and guide decision-making and to guide studies that have greater relevance and advanced methodologies. Besides, even with ineffective or insufficient results, there is a generation of production of scientific evidence, which reduces uncertainties and doubts and encourages the emergence of alternative hypotheses. Negative outcomes are scientifically documented, which is also important for research.

The results of the thematic evolution structure (see Figure 5, above) stressed aspects concerning how the themes of the research field focused on COVID-19, developed over the period explored (January to August 2020). This map provides the possibility of raising a hypothesis: would humanity be condemned to suffer other illnesses caused by devastating viral loads as serious as that of Sars-Cov-2? Would large populations be prepared for a united solidarity action with the purpose of containing the virus? Punctual answers to such questions, for now, are not possible. However, it is clear that the history of civilization was marked, at other times, by similar phenomena, which were reversed due to the uninterrupted efforts focused on the development of scientific knowledge.

In view of the potential strength of the virus to spread worldwide, countless attempts to contain it have failed. As the main associated factor, it is pointed out the stimulus, on the part of governments, of economic and social activities, making possible breaches of emergency protocols, such as the lockdown. It is understood that measures like this, aim exclusively at the safety of the population, since, avoiding the collapse in health systems, researchers have the time available to understand the effects caused by Sars-Cov-2, in ways how it operates and with that, look for a solution, that is, an immunizing vaccine. In the midst of chaos, subjects from all walks of life are conditioned to rethink personal and collective concepts. Experiencing everyday life, now permeated by the unknown and invisible, sometimes generates anxiety and depression. Such aspects are also related to the current economic crisis, clarifying the values of capitalist productive models, which placed the mark, the right to life of any and all citizens.

## 6. Conclusions

In this research a BNA was performed in order to investigate the strategic themes and the thematic evolution structure of COVID-19 during the first eight months. Although there are several bibliometric analyses on the coronavirus, they focus on different strains of the virus that emerged between 1970 and 2020 (see Table 1). In this BNA, the focus is the pandemic caused by the coronavirus in 2020 and the relevant related themes. The analysis takes place under an interdisciplinary view, based on the human, biological and exact sciences, which enables a broader and deeper understanding of the pandemic impacts for individuals. Our results highlighted the strategic themes such as ‘CLOROQUINE’, ‘ANXIETY’, ‘VACCINE’, among others, as well as its thematic network structure, to understand and find hidden patterns. The evolution structure showed how the themes of COVID-19 research field are evolving over time. This evolution map shows several challenges that scientific community are trying to overcome such as comorbidities and diseases caused by COVID-19 and damage prevention and containment of the virus. Such results presented trends of COVID-19 and possible scenarios of research hotspots. The relationship between the themes, understood from the details of the networks, allows us to view the concerns and reflect on the tireless attempts of health professionals and academics to slow down the spread of Sars-Cov-2 and to promote resilience systems and treatment possibilities.

It is reasonable to suggest future works related to psychological aspects in order to deal with coronavirus consequences, especially regarding to depression and anxiety aspects. Future works can be related to strategic themes, however, is vital to perform researches in fields with few development such as Q2 and Q3 in order to avoid mitigation of immature themes. The limitations of this work must also be highlighted: only WoS was used to perform this research and articles using SciMAT. Despite the fact WoS indexes journals with higher impact factor [120] this is not a determinant factor by itself, because WoS is also behind Scopus in in terms of number of journals indexed and the speed of citations analysis [120], which shows that some relevant researches may be left out. Moreover, studies argue that WoS is not the easiest and most attractive database to work with at first impression, if compared to Scopus [120]. Its field also has limitations when favor natural sciences and engineering, that represents 43% of the documents, and biomedical research with 27%, compared to 21% of social sciences and 9% of arts and humanities, still underrepresented. At the same way, English-language journals are overrepresentation to the detriment of other languages [121,122]. Therefore, future works must be conducted in order to investigate other databases such as Scopus, PubMed, Science Direct, etc. Other bibliometric tools such as VOSviewer, CiteSpace, Sci2tool, among others, should also be used in other to collect different point of views which will help to advance the field of COVID-19. The results will form the basis for future research and guide decision-making in COVID-19 research and treatments.

## Figures and Tables

**Table 1:** Existing BNA of COVID-19 in the Web of Science.

Study	Coverage	Focus
[12]	January–April 2020	Analysis of works developed on COVID-19 by BNA to discuss epidemiological trends using VOSviewer software
[13]	1968–April 2020	Identification of macro-level aspects from the scientometric analysis of COVID-19 in the literature using VOSviewer software.
[14]	January–July 2020	Review of publications and the general trend of COVID-19 by Iranian scientists using VOSviewer software.
[15]	January–May 2020	Keywords clustering of the COVID-19 research field using the VOSviewer Tool.
[16]	January–March 2020	Review of articles in English to assess the scientific response to the COVID-19 Pandemic by conducting a bibliometric survey using the arXiv, bioRxiv, medRxiv and MDPI Preprints databases.
[17]	2019–March 2020	Bibliometric analysis to compare COVID-19 research between English and Chinese-language journals using VOSviewer and CiteSpace Software.
[18]	January–July 2020	Identification of the status of documents published in nursing journals on COVID-19.
[19]	December 2019–June 2020	Evaluation of the global scientific production of COVID-19 research by bibliometric analysis to determine the most cited publications and explore current topics using VOSviewer.
[20]	2015–2020	Analysis of scientific globalism about COVID-19 and non-COVID-19 articles during the pandemic and 5 years before the pandemic period using scientometric analysis.
[21]	January–March 2020	Depiction of the growth of medical literature on COVID-19 using evidence maps and bibliometric analysis to identify gaps in research in the early stage of the pandemic using Python software.
[22]	1970–March 2020	Bibliometric analysis of articles cited in the COVID-19 research published until 2019 and articles published in a pandemic situation using VOSviewer software.
[23]	2019–May 2020	Presentation of a broad understanding of COVID-19 and directions for future research through bibliometric analysis using VOSviewer software.
[24]	November 2019–March 2020	Exploration of the responses to COVID-19 and definition of challenges during the initial phases of the pandemic through a bibliometric and transversal review of the literature.
[6]	1945–2020	Bibliometric analysis of the most productive countries in coronavirus publications and international scientific collaboration and open accessibility typology for these publications using VOSviewer.
[25]	January–April 2020	Evaluation of the information flow quality and scientific collaboration using RISmed R package and a custom Python script available on GitHub.
[7]	2000–April 2020	Depiction of the scientific response to international public health emergencies in a comparative bibliometric study of various outbreaks using VOSviewer software.
[26]	2003–2020	Analysis of the coronavirus literature published since the SARS outbreak in 2003 by bibliometric analysis using Citespace and VOSviewer software.
[27]	January–May 2020	Investigating coverage of publications related to COVID-19 in India using VOSviewer to identify authors, institutes, international collaboration, keywords and journals preferred by Indian researchers.
[28]	2003–2020	A quantitative and qualitative analysis of the knowledge base and topics of coronavirus research to provide an overview of the publications with the use of CiteSpace.
[29]	January 2003–February 2020	Assessing coronavirus status and research trends worldwide to find out what topics are popular for researchers interested in coronavirus using VOSviewer software.
[30]	December 2019–April 2020	Application of machine learning bibliometric analysis on COVID-19 publications to identify trends and guide future research using R-Studio software.
[31]	1970–2020	Analysis of the development of research topics on coronavirus and the main related concepts available in the literature using techniques and bibliometric tools with the SciMAT software.
[32]	January 2000–March 2020	Bibliometric analysis to assess the impact of coronavirus research on global scientific production and the contribution to the prevention and control of COVID-19 using CiteSpace software.
[8]	2000–February 2020	Analysis of previous coronavirus research to observe future research points and provided an in-depth bibliometric analysis of COVID-19 using VOSviewer software.
[33]	January April 2020	Global view on the daily growth of scientific production on COVID-19 in different sources of information.
[34]	January–March 2020	Analysis of publications on COVID-19 to summarize research hotspots and conduct a bibliometric review to serve as a basis
[35]	2003–2020	Sought to find research, flows and themes using the coronavirus literature in the social sciences field using ‘biblioshiny’ the web-based interface of the R-package (Bibliometrix 3.0).
